# COVID-19 preventive practices and associated factors among high school and preparatory school students in Dessie City, Ethiopia

**DOI:** 10.3389/fpubh.2022.1019584

**Published:** 2022-11-21

**Authors:** Alelgne Feleke, Mesfin Gebrehiwot, Helmut Kloos, Asha Embrandiri, Chala Daba, Seada Hassen, Metadel Adane

**Affiliations:** ^1^Department of Environmental Health, College of Medicine and Health Sciences, Wollo University, Dessie, Ethiopia; ^2^Department of Epidemiology and Biostatistics, University of California, San Francisco, San Francisco, CA, United States

**Keywords:** COVID-19, preventive practices, high school and preparatory school students, Ethiopia, infection prevention and control

## Abstract

**Background:**

As the COVID-19 pandemic continues to ravage the world, the most pretentious sector besides the economy is the education system. Ethiopia is not equipped with the infrastructure and facilities to provide online classes for students at all levels. Hence, all institutions were re-opened with mandatory infection prevention and control (IPC) protocols such as the use of face masks, physical distancing, shifts in classes, and routine hand washing practices with soap and water to restrict the spread of the virus. Nevertheless, there has been no monitoring and follow- up and there is no data on IPC compliance among school children in the country. The purpose of this study was to examine the COVID-19 preventive practices and their associated factors among high and preparatory school students in Dessie City, Ethiopia.

**Methods:**

A cross-sectional study was carried out by using a pre-tested face-to-face applied structured questionnaire and direct observations from March 8 to March 20, 2021, in five high and preparatory schools in Dessie City. The sample size was proportionally allocated in each school based on the students' total number registered in the first academic semester, then stratified by grade level, and proportionally allocated to each grade and section. Finally, a simple random sampling method was used to select study participants. Variables with *p-*values < 0.25 in the bivariate logistic regression analysis were entered into the multivariate logistic regression model.

**Results:**

This study involved 422 students with a response rate of 98.8%. The level of good preventive practices was 43.7%. After adjusting for covariates, female, positive attitudes, received IPC training and clear accessible sharing of information and feedback with parents, students and teachers were identified as predictors of good precaution practices.

**Conclusion:**

The prevalence of good prevention practices for COVID-19 among students was relatively very low. Therefore, the Department of Health and Education of Dessie City and each school should implement environmental health programs and promote prevention practices in high schools and preparatory schools.

## Introduction

COVID-19, caused by severe acute respiratory syndrome-coronavirus 2 (SARS CoV-2 virus) was first detected in Hubei Province of China in December 2019 (1, 2). The contagious nature of the virus led WHO to declare it in March 2020 as a global pandemic (3, 4). By September 17, 2022, the virus had infected 493,429 people in Ethiopia (5). The most common initial COVID-19 symptoms include fever, dry cough, fatigue, breathing difficulties, and diarrhea (6, 7). These symptoms are normally mild, develop gradually and sometimes lead to serious complications such as cardiac and respiratory failures and death, especially in patients with comorbidities and in older people (8). The manifestation and symptoms change with new emerging variants of the virus (9). The virus is transmitted from person to person by respiratory droplets during sneezing, coughing, talking (10, 11) and through contact with people and surfaces (12, 13). The pandemic has spread at a slower pace in Africa than in other parts of the world (14, 15), apparently due to previous exposure of Africans to other infectious diseases such as Ebola virus disease and measles and the relatively young African population (15). However, due to lack of infrastructure, qualified personnel, and other resources, African health care systems experienced major difficulties in providing care for infected persons (16). Owing to the scarcity of vaccines, preventive measures promoting behavioral change continue to be more commonly used than vaccines in Africa, in contrast to Europe, East Asia, the USA and other industrialized countries (17).

After the outbreak of COVID-19 in early 2020, WHO proposed effective IPC, including the use of face masks and other personal protective equipment (PPE), alcohol-based sanitizers, social distancing, regular hand washing, contact tracing, and quarantine (18). Compliance with IPC protocols have been difficult in minimizing the risk of virus transmission (19) and the COVID-19 pandemic has disrupted public education (20). Stay-at-home pronouncements caused the closure of educational institutions across the world, prompting the shift to online teaching. Ethiopia, one of the least developed countries, does not have the resources for distant learning (13). As the schools and families do not have the resources for distant learning, the Ethiopian government recommends starting the teaching and learning processes in schools by implementing the COVID-19 prevention protocol. Hence, the government instructed all Ethiopian institutions to continue classroom teaching with the IPC directives. However, there is no detailed information on the enforcement, monitoring and compliance with these regulations and no COVID-19 prevention studies have been carried out in schools. This study examines the preventive practices and their associated factors for students in five high schools and preparatory schools in Dessie City, Ethiopia.

## Materials and methods

### Study area and study design

A cross-sectional study was carried out by using a pre-tested face-to-face applied structured questionnaire and direct observations from March 8 to March 20, 2021 in the five schools in Dessie City. Dessie is located in the highlands of Amhara Region. Of the city's five high schools and preparatory schools, three were government schools and two were private schools, with 9,024 students (4,341 males and 4,683 females) enrolled in March 2021.

### Source population, inclusion and exclusion criteria

All Dessie City students in grades 9 through 12 enrolled from March 8 to March 20, 2021 were the source population and the sampled students who were available throughout the study period and willing to take part in the research were the study population. Students who were absent during the data collection period between March 8 and March 20 were excluded from the study.

### Sample size determination

A single population proportion formula was used to obtain the sample size for this study.

The following assumptions were made: a 50% prevalence of preventive practices of students in high and preparatory schools since no study in Ethiopia on school students, a margin of error of 5%, a 95% CI, and a 10% non-response rate.


$$\begin{array}{l} {\left[\text{Z}\alpha /2\right]}^{2^{*}}\text{P}\left[1-\text{P}\right]/d^{2}=n \end{array}$$

By adding a 10% non-response rate, the final sample size was 422 students.

### Sampling technique

The sample size was allocated proportionally in each of the five schools studied based on the number of students enrolled in the first academic semester. The samples were then proportionally allocated to the 9^*th*^, 10^*th*^, 11^*th*^, and 12^*th*^ grade levels, with each component of the relevant grade levels being further proportionally allocated. Finally, study participants were chosen by random sampling (lottery method) with classroom attendance as the sampling frame at the time of the survey.

### Data collection methods and quality assurance

The face-to-face administered tools were adapted from the literature, the 2016 Ethiopian Demographic Health Survey (21), WHO (22), and UNICEF reports (23). Data were collected using structured questionnaire (self-reporting) and direct observation. The questionnaire consisted of five main sections: Section A contained 10 questions about socio-demographic status and sources of information on COVID-19; Section B contained 14 questions about water, sanitation, and hygiene behavior; Section C contained 17 questions about knowledge; Section D contained 15 questions about attitude, and Section E contained 22 questions about practice.

The data collectors were three environmental health professionals and three clinical nurses. One data collector was recruited for each school. The tool was prepared in English and then translated to Amharic, the local language, and back to English by two translators to check its consistency and completeness. The 2 days of training focused on instructing data collectors on how to collect data. The validity of the questionnaire was assessed by pretesting with 21 (5%) high and preparatory school students in nearby Kombolcha Town, and some amendments such as question order, avoiding less important questions, and editing questions that are unclear were made before the survey.

The reliability was determined by calculating the Cronbach's alpha method, which demonstrated satisfactory internal consistency. The Cronbach's alpha value for the preventive practice was 0.79, which indicates high reliability. No personal identifiers were obtained on the day of data collection, and COID-19 prevention measures were implemented throughout the procedure. The principal investigator supervised the process of data collection and supported the data collectors. Each questionnaire was checked daily for completeness and consistency before entering into computer data collectors. To check the quality of the entered data, 10% of the entered questionnaires were randomly selected and re-entered to identify potential data entry errors.

### Outcome and explanatory variables

The outcome variable was preventive practices (good or poor) and the explanatory variables were socio-demographic status, water sanitation, hygiene (WASH), and sources of COVID-19 information. Four basic divisions were used to organize the explanatory variables. Section 1: Socio-demographic variables and source of information include age, sex, religion, grade, and household size, updated information from television, face book, internet Wi-Fi, families/friends, and received IPC training. Section 2: WASH variables; They include' number of students per class room, ventilation of class rooms and toilets, availability of alternative classes, posting of COVID-19 information in schools, sharing of information and feedback mechanisms between parents, students and teachers, information posts for events related to COVID- 19 in the schools, availability of psychosocial support, availability of piped water, availability of hand washing facilities, and availability of soap and alcohol-based hand sanitizer near wash basins. Section 3: Knowledge about coronavirus disease and its prevention and Section 4: Attitudes toward COVID-19 prevention.

### Operational definitions

**High school students**: Students in grades 9 and 10 in 2020/21.

**Preparatory school students**: Students in 11^*th*^ and 12^*th*^.

**Knowledge**: Respondents who scored equal to or above the mean value (≥14 out of 17 questions), were considered as having good knowledge, whereas a score of less than the mean value was considered as having poor knowledge about COVID-19 precautionary measures (Appendix 1).

**Attitudes:** Respondents who scored equal to or above the mean value (≥10 out of 15 questions), were considered to have a positive attitude, and scores of less than the mean value were considered to have a negative attitude about COVID-19 prevention (Appendix 1).

**Practices:** Respondents who scored equal to or above the mean value (≥11 out of 22 questions), were considered as having good preventive practices, whereas scores less than the mean value were classified as poor practices (see **Table 4**).

### Data management and analysis

Prior to exporting the data to SPSS version 25.0 for analysis, it was cleaned, coded, and entered into EpiData version 3.1. Descriptive statistics were employed to evaluate the overall distribution, including means with standard deviations for continuous variables and frequencies and percentages for categorical variables. A binary logistic regression model with a 95% confidence interval was used to analyze the data (CI). A bivariate logistic regression analysis [crude odds ratio (COR)], as well as a regression of multivariate logistic analysis [adjusted odds ratio (AOR)], were carried out. Variables having a *p* < 0.250 were chosen for the analysis of multivariate logistic regression from the bivariate analysis (24). Variables having a significance level of <0.05 from the analysis of multivariate logistic regression were considered statistically significant and independently linked with good preventive measures among students.

Standard error with a cut-off value of 2 was used to check for multi-collinearity among independent variables. We discovered that the greatest standard error was 0.839, indicating that there was no multi-collinearity. The Hosmer Lemeshow test (24) was used to assess model fitness, yielding a *p*-value of 0.347, indicating that the model was fit.

## Results

### Socio-demographic characteristics of participants

Of the 422 students selected, 417 participated in the study, with a response rate of 98.8%. More than half of the students were Orthodox Christians [245 (58.8%)] and 89.4% of the participants were between 15 and 18 years old. The mean [±SD (standard deviation)] age of the students was 16.97 (±1.28), three-fifths [256 (61.4%)] were females and almost all (402, 96.4%)] were single. Nearly three-fourths (65.2%) of the participants were living in households with 4–6 members. Almost all participants got updated information on COVID-19 from television (377, 90.4%), families/and friends (395, 94.7%) (Table 1).

**Table 1:** Socio-demographic status and source of COVID-19 information utilized by high school and preparatory school students in Dessie City, Northeast Ethiopia.

**Independent variables**	**Category**	**Frequency (*n* = 417)**	**Percentage (%)**
Age	10–14 years	12	2.9
	15–18 years	373	89.4
	≥19 years	32	7.7
Sex	Male	161	38.6
	Female	256	61.4
Religion	Orthodox Christian	245	58.8
	Muslim	172	41.2
Grade	9	50	12.0
	10	115	27.6
	11	54	12.9
	12	198	47.5
Household size	1–3	36	8.6
	4–6	272	65.2
	>6	109	26.1
Get updated information from television	Yes	377	90.4
	No	40	9.6
Get updated information from Facebook	Yes	331	79.4
	No	86	20.6
Get updated information from the internet or Wi-Fi	Yes	356	85.4
	No	61	14.6
Get updated information from families/friends	Yes	395	94.7
	No	22	5.3
Received IPC training	Yes	190	45.6
	No	227	54.4

### WASH and preventive behavior

The main water source at all the schools was piped water in the school yards. There was no water interruption during the 2 weeks preceding the study (Table 2). The schools also posted some COVID-19 related information in its compounds (Table 3). There were hand-washing facilities in all five schools but no water and soap, and alcohol-based hand sanitizers were not available in any of the five schools during the observation period. Three-quarters (75.1%) of the students said that their classes had 39 or more students (Table 2), resulting in crowded conditions.

**Table 2:** Water, sanitation, and hygiene practices at high school and preparatory school students in Dessie City, Northeast Ethiopia.

**Independent variables**	**Category**	**Frequency (*n* = 417)**	**Percentage (%)**
Number of students in your	<39	104	24.9
class?	≥39	313	75.1
Are classrooms well-ventilated (open windows)?	Yes	417	100
Are classrooms well-ventilated	Yes	417	100
Is piped water available in this school?	Yes	417	100
Was the water supply interrupted in this school during the last 2 weeks?	No	417	100
Are hands washing facilities available in this school?	Yes	417	100
If yes, are soap and water available at the wash basins?	No	417	100
Is alcohol-based hand sanitizer available at the wash basins?	No	417	100
Are the toilets well-ventilated?	Yes	417	100

**Table 3:** COVID-19 related information posted at high schools and preparatory schools in Dessie City, Northeast Ethiopia.

**Independent variables**		**Frequency (*n* = 417)**	**Percentage (%)**
Is information on COVID 19	Yes	262	62.8
posted in the school?	No	155	37.2
Is an emergency telephone for	Yes	62	14.9
COVID-19 available in the school?	No	355	85.1
Is there sharing of information	Yes	206	49.4
and feedback mechanisms	No	211	50.6
between parents, students and teachers?			
Is information provided on	Yes	191	45.8
events related to COVID 19 in this school?	No	226	54.2
Is there psychosocial support for	Yes	179	42.9
COVID-19 cases in this school?	No	238	57.1

More than half (247, 59.2%) of the students said that they always washed their hands using soap and water for a minimum of 20 s. Similarly, 218 (52.3%) allegedly used alcohol-based hand sanitizers. One-fourths (102, 24.5%) of the study participants washed their hands before entering the classroom, 94 (22.5%) before putting on and 92 (22.1%) after taking off a facemask and after sneezing and coughing (Table 2). Two-thirds (279, 66.9%) of the students supposedly wore a face mask or face covering cloth every time they left their home. Of the 417 participants, 145 (34.8%) stated that they always tried to maintain a 2- meter social distance when in public areas. Most of the study participants (345, 82.7%) had not reduced visits with friends and families, 254 (60.9%) continued to make non-essential trips outside their home and 238 (57.1%) and had no aversion to visiting crowded places. Nearly two-fifths (161, 38.6%) of the students avoided shaking hands, hugging and kissing during greetings since the advent of the coronavirus pandemic (Table 4).

**Table 4:** COVID-19 prevention measures among high school and preparatory school students in Dessie City, Northeast Ethiopia.

**Practice question**	**Response**	**Frequency**	**Percentage (%)**
Do you always wash your hands with soap and water for at least 20 s?	Yes	247	59.2
	No	170	40.8
Do you wash your hands	Yes	137	32.9
before contact of things?	No	280	67.1
Do you wash your hands after	Yes	176	42.2
contact with any surface?	No	241	57.8
Do you wash your hands if	Yes	317	76.0
you look or feel dirty?	No	100	24.0
Do you wash your hands	Yes	102	24.5
before entering the classroom?	No	315	75.5
Do you wash your hands after	Yes	109	26.1
exiting the classroom?	No	308	73.9
Do you wash your hands after	Yes	283	67.9
use of the toilet?	No	134	32.1
Do you wash your hands	Yes	106	25.4
after playing?	No	311	74.6
Do you wash your hands after	Yes	92	22.1
sneezing/coughing?	No	325	77.9
Do you wash your hands after	Yes	107	25.7
touching public goods, including money?	No	310	74.3
Do you wash your hands after	Yes	123	29.5
coming home from school and other public places?	No	294	70.5
Do you wash your hands	Yes	94	22.5
before and after wearing	No	323	77.5
a mask?			
Do you frequently use	Yes	218	52.3
alcohol-based hand sanitizer?	No	199	47.7
Do you always wear a face	Yes	279	66.9
mask/face covering cloth every time you leave your home to prevent COVID-19?	No	138	33.1
Do you throw used tissues	Yes	311	74.6
after blowing your nose in the trash?	No	106	25.4
Do you always cover your	Yes	272	65.2
mouth and nose with an elbow when coughing and sneezing?	No	145	34.8
Do you always avoid touching	Yes	227	54.4
your nose, mouth and eyes with unwashed hands?	No	190	45.6
Do you always avoid shaking	Yes	161	38.6
hands, hugging and kissing during greetings?	No	256	61.4
Do you always keep a social	Yes	145	34.8
distance of at least 2 meters?	No	272	65.2
Do you always avoid	Yes	163	39.1
non-essential trips outside your home?	No	254	60.9
Do you always avoid going to	Yes	179	42.9
crowded places?	No	238	57.1
Do you always avoid meeting	Yes	72	17.3
with friends and relatives?	No	345	82.7

Overall, more than half (237, 56.8%) (95% CI: 52.3–61.9) of the students revealed that they had poor preventive practices and only 180 (43.2%) (95% CI: 38.1–47.7) had good practices (Table 4).

### Factors associated with COVID-19 prevention

In the multivariable analysis, females, having received IPC training, sharing information and feedback established by parents, students and teachers who had a positive attitude were significantly associated with good preventive practices. Good preventive measures were 1.96 times more prevalent in females than in males (AOR = 1.96, 95% CI [1.24–3.10]). Students who received IPC training were 1.91 times more likely to practice prevention than those without training (AOR = 1.91, 95% CI [1.23–2.96]). Those who shared information and feedback with parents and teachers had a 2.11 times higher preventive practices score (AOR = 2.11, 95% CI [1.37–3.25]), and those with a positive perception of coronavirus precautionary measures were 3.33 times more likely to properly practice preventive practices (AOR = 3.33,95% CI [2.10–5.27]) than their counterparts (Tables 5, 6).

**Table 5:** Bivariate analysis of socio-demographic characteristics and sources of information on COVID-19 at high schools and preparatory schools in Dessie City, Northeast Ethiopia.

**Variables**	**Category**	**Practice**		**COR (95% CI)**	***P*-value**
		**Good**	**Poor**		
Age	10–14 years	7	5	Ref.	
	15–18 years	167	206	0.58 (0.18–1.86)	0.358
	≥19 years	6	26	0.17 (0.04–0.700)	0.015
Sex	Male	109	52	Ref.	
	Female	128	128	2.09 (1.39–3.16)	0.000
Religion	Orthodox	100	145	Ref.	
	Muslim	80	92	1.26 (0.85–1.87)	0.248
Grade	9	21	29	Ref.	
	10	48	67	0.99 (0.51–1.94)	0.975
	11	21	33	0.88 (0.40–1.93)	0.747
	12	90	108	1.15 (0.61–2.16)	0.661
Household size	1–3	9	27	0.49 (0.21–1.15)	0.101
	4–6	127	145	1.29 (0.82–2.03)	0.263
	>6	44	65	Ref.	
Do you get COVID-19 information from television?	Yes	164	213	0.87 (0.45–1.68)	0.671
	No	16	24	Ref.	
Do you get COVID-19 information from Facebook?	Yes	147	184	0.78 (0.48–1.270	0.314
	No	33	53	Ref.	
Do you get COVID-19 information from online sources?	Yes	154	202	0.97 (0.56–1.69)	0.926
	No	26	35	Ref.	
Do you get COVID-19 information from your families/or friends?	Yes	167	215	0.76 (0.37–1.56)	0.453
	No	13	22	Ref.	
Did you receive IPC training?	Yes	101	89	0.47 (0.32–0.69)	0.000
	No	79	148	Ref.	
Number of students in your class	<39	56	48	0.56 (0.36–0.88)	0.012
	≥39	124	189	Ref.	
Is information about COVID-19 transmission posted in the school?	Yes	118	144	0.81 (0.54–1.22)	0.316
	No	62	93	Ref.	
Is information and feedback mechanisms shared between parents, students and teachers?	Yes	112	94	0.40 (0.2–0.59)	0.000
	No	68	143	Ref.	
Is information on COVID-19 regularly provided in the school on events related to COVID 19?	Yes	87	104	0.84 (0.57–1.23)	0.366
	No	93	133	Ref.	
Is there psychosocial support for COVID-19 cases in this school?	Yes	76	103	1.05 (0.71–1.56)	0.800
	No	104	134	Ref.	
Overall knowledge	Good	143	169	1.56 (0.98–2.46)	0.059
	Poor	37	68	Ref.	
Overall attitude	Positive	136	115	3.28 (2.14–5.02)	<0.001
	Negative	44	122	Ref.	

Ref., reference category.

**Table 6:** Multivariate study of socio-demographic characteristics and sources of information regarding COVID-19 at high schools and preparatory schools in Dessie City, Northeast Ethiopia.

**Variables**	**Category**	**Practice**		**AOR** **(95% CI)**	***P*-value**
		**Good**	**Poor**		
Sex	Male	109	52	Ref.	
	Female	128	128	1.96 (1.24–3.10)	0.004
Did you receive IPC training?	Yes	101	89	1.91 (1.23–2.96)	0.004
	No	79	148	Ref.	
Is information and feedback	Yes	112	94	2.11 (1.37–3.25)	0.001
mechanisms shared between	No	68	143	Ref.	
parents, students and teachers?					
Overall attitude	Positive	136	115	3.33 (2.10–5.27)	<0.001
	Negative	44	122	Ref.	

Ref., reference category.

## Discussion

The low prevalence (43.2%) of good preventive practices for COVID-19 among students in Dessie City is similar as in other studies in Ethiopia (25–28), Bangladesh (29), Sudan (30), and Egypt (31) and is lower than in other studies conducted in Ethiopia (32–35), Nepal (36), Syria (37), China (38), Uganda (39), and Pakistan (40). These discrepancies may be caused by variations in socio-demographic characteristics of the study populations, study areas, the time studies were carried out, occupation (health professionals vs. students), and the effectiveness of local COVID-19 programs. Probable reasons for the low preventive practices in this study may include the absence of an effective and comprehensive COVID-19 prevention program in the schools.

The higher prevalence of good preventive practices in Dessie schools than in three other communities in Ethiopia (41–43) is difficult to explain because they were carried out in general populations and among healthcare workers.

In this study, 247 (59.2%) participants allegedly washed their hands for a minimum of 20 s using soap and water, corroborating findings in Pakistan (56.0%) (44). But this rate is lower than those reported by two other studies in Ethiopia (81.4%) (32), (77.3%) (44) and studies in Pakistan (85.5%) (45), (94.0%) (46), Iran (82.2%) (47) and Beijing, China (91.3%) (48). These high rates were due to the relatively high socioeconomic level of participants, good WASH facilities, commitment of the government, and the recent study periods. The proportion of students washing their hands using soap and water after contacting any surface (176, 42.2%) was similar in our study as the one by Gebretsadik et al. among hospital visitors in another Ethiopian community (48.1%) (44).

The proportion of students reportedly wearing face masks when leaving home (66.9%) was similar to that reported by Jemal et al. (67.3%) (33) among Ethiopian health care workers and internet users in Nigeria (65.0%) (45), and high school students and young adults in Bangladesh (70.6%) (46). But our rate was higher than those reported for hospital patients (28) and a general population in Ethiopia (47), shoppers in the USA (41.0%) (48), a rural population in Egypt (47.3%) (49), and a general population in Syria (27.9%) (37). These variations may be due to differences in study dates and the effectiveness of local COVID-19 programs.

Only 38.6% of the students avoided shaking hands, hugging and kissing during greetings, a lower rate than those reported for Ethiopian health care workers (50), hospital visitors (48), and a predominantly rural population (44) and Syrian youth and adults on a social platform (92.5%) (37). Although the heterogeneity of these populations makes comparisons difficult, the absence of a COVID-19 prevention program in Dessie schools appears to be a significant factor in their low rates.

The rate of reporting good preventive practices was 1.9 times higher in females than in males. Similar ratios were reported by three other studies in Ethiopia (42, 51, 52) and by studies in Bangladesh (53), Uganda (54), Syria (37), Jordan (55), Iran (56), Beijing (57), Bangladesh (29), and in Saudi Arabia (58).

The positive attitude of the students about coronavirus prevention 3.33 times had better preventive practices than those who had a negative attitude. Similar rates were reported by a study in Sidama Region, Ethiopia (41), in northwest Ethiopia (52), Karachi, Pakistan (59), and north-central Nigeria (60), among primary, middle school students (57), and university students of China (38) and in the Sudanese population (30).

Students who had received IPC training were 1.91 times more likely to mention good preventive practices than those without IPC training, corroborating a study on health workers in Uganda (54) and frontline health workers in Nepal (61).

## Study limitations

One possible limitation is that some students may appear to have given socially desirable response, mostly to questions about preventive behavior, although the extent of social desirability bias could not be determined.

## Conclusion

This study revealed low rates of preventive behavior for COVID-19 and a wide gap between students' knowledge and practices. Scarcity of sanitary media, absence of accessible prevention and control training programs, and failure of parents and students to share information were major factors in the low prevention rates. We recommend that the Health and Education departments of Dessie City and administrations of the high schools and preparatory schools implement comprehensive COVID-19 prevention programs that address deficiencies in environmental health and promote health-enhancing behavior and attitudes. Furthermore, these entities should provide free personal protective equipment such as facemasks and alcohol-based hand sanitizer.

## Data availability statement

The raw data supporting the conclusions of this article will be made available by the authors, without undue reservation.

## Ethics statement

The studies involving human participants were reviewed and approved by Ethical Review Committee of Wollo University's College of Medicine and Health Sciences gave their approval (Protocol number: CMHS/146/03/2021). Written informed consent to participate in this study was provided by the participants' legal guardian/next of kin.

## Author contributions

All authors made a significant contribution to the work starting from the conception, study design, execution, acquisition of data, analysis and interpretation, in drafting, revising, or critically reviewing the manuscript. The authors also gave final approval of the version to be published, have agreed on the journal to which the article has been submitted, and agree to be accountable for all aspects of the work.

## Conflict of interest

The authors declare that the research was conducted in the absence of any commercial or financial relationships that could be construed as a potential conflict of interest.

## Publisher's note

All claims expressed in this article are solely those of the authors and do not necessarily represent those of their affiliated organizations, or those of the publisher, the editors and the reviewers. Any product that may be evaluated in this article, or claim that may be made by its manufacturer, is not guaranteed or endorsed by the publisher.

## Abbreviations

AOR: Adjusted odds ratio
COR: Crude odds ratio
IPC: Infection prevention and control
WASH: Water, sanitation and hygiene.
