# Supplementary material for: COVID-19 preventive practices and associated factors among high school and preparatory school students in Dessie City, Ethiopia
Source: Front Public Health. 2022 Nov 21;10:1019584. doi: 10.3389/fpubh.2022.1019584 (PMC9720378; doi:10.3389/fpubh.2022.1019584)
Supplement: Supplementary file 1 [file Table_1.DOCX]

**Preventive practices for COVID-19 and associated factors among high school and preparatory school students in Dessie City, northeastern Ethiopia**

**Alelgne Feleke^1*^, Mesfin Gebrehiwot^1*^, Helmut Kloos^2^, Asha Embrandiri^1^, Chala Daba^1^, Seada Hassen^1^, Metadel Adane^1*^**

^1^Department of Environmental Health, College of Medicine and Health Sciences, Wollo University, Dessie, Ethiopia

^2^Department of Epidemiology and Biostatistics, University of California, San Francisco, USA

**Appendix 1**

**Table 1. Knowledge of high school and preparatory school students towards COVID-19 transmission and prevention in Dessie City, northeast Ethiopia.**

| Knowledge questions |
| --- |
| Is COVID-19 transmitted by air droplets? |
|  |
| Is COVID-19 transmitted by physical contact? |
|  |
| Is COVID-19 transmitted by evil spirits? |
|  |
| Are all people at risk of COVID-19? |
|  |
| Can the virus be transferred by shaking hands? |
|  |
| Can touching your nose, mouth, eyes with unwashed hands transfer the virus? |
|  |
| Can proper washing of hands for at-least 20 seconds with soap and water prevent COVID-19? |
|  |
| Can rubbing of hands with alcohol- based sanitizer prevent transmission of COVID-19? |
|  |
| Is there a need to wash hands before and after touching any surface using water and soap or sanitizer? |
|  |
| Can sneezing or coughing into your arm/elbow prevent the spread of the virus? |
|  |
| Can staying at home decrease the chance of getting infected? |
|  |
| Can distancing 2 meters between people prevent the transmission of COVID-19? |
|  |
| Does wearing a face mask decrease the risk of transmission of COVID-19? |
|  |
| Can cloth masks be reused after washing with soap and water? |
|  |
| Avoiding crowded places reduces the transmission of corona virus |
|  |
| Does wearing gloves replace the need for hand washing or use of alcohol -based hand sanitizer? |
|  |
| Can isolating oneself if getting sick prevent the spread of the virus? |
|  |

**Table 2. Attitudes of** **high school and preparatory school students towards COVID-19 prevention in Dessie City, northeast Ethiopia.**

| Attitude questions |
| --- |
|  |
| Can the COVID-19 epidemic be controlled in Dessie? |
|  |
| Do you think the epidemic can be controlled by people following all guidelines by the Ministry of Health? |
|  |
| Do you think the epidemic can be controlled with drugs? |
|  |
| Do you think the government should play a role in the prevention and control of COVID-19? |
|  |
| Do you think the schools should play a central role in the prevention and control of COVID-19? |
|  |
| Do you think each person should play a major role in the prevention and control of COVID-19? |
|  |
| Do you think traditional medicine should be used in the prevention and control of COVID-19? |
|  |
| Are prayers effective to prevent COVID-19? |
|  |
| Do you think that you can contribute to preventing and controlling the COVID-19 epidemic? |
|  |
| Do you believe that frequent hand washing damages skin and causes cracking, dryness, irritation and dermatitis? |
|  |
| Do you believe you can protect yourself against COVID-19? |
|  |
| Do you believe you have a very low risk of acquiring COVID-19 from others? |
|  |
| Listening and following the direction of state and local authorities reduces COVID-19 transmission? |
|  |
| Do you worry about contracting COVID-19? |
|  |
| Do you think that the disease causes embarrassment or insult to infected persons or the relatives of students? |
|  |
